# Conscious and unconscious processes in vision and homeostasis

**DOI:** 10.3389/fnbeh.2025.1516127

**Published:** 2025-02-07

**Authors:** Athanassios S. Fokas, Nikos K. Logothetis

**Affiliations:** ^1^Department of Applied Mathematics and Theoretical Physics, University of Cambridge, Cambridge, United Kingdom; ^2^International Center for Primate Brain Research, Chinese Academy of Sciences, Shanghai, China

**Keywords:** mental image, unconscious-conscious continuum, binocular rivalry, visual bi-stability, emotions-feelings

## Abstract

The central and autonomic communications affecting cognition, emotions, and visceral functions, are determined by the interaction of unconscious and conscious processes. In this regard, we discuss two basic hypotheses. First, *unconscious and conscious processes form a dynamic organizational continuum*. Second, *emotions, which are unconscious forms of feeling, characterize the response of an organism to any internal change disturbing homeostasis or to any change in the exterior of the organism detected via specialized sensory probes*. The former hypothesis is illustrated by discussing aspects of visual perception. The validity of the second hypothesis is supported by discussing interactions between unconscious and conscious process necessary for maintaining energy and water balance.

## 1 Introduction

*The first hypothesis* implies that *every conscious experience is preceded by an unconscious process*. Yet, an attempt to fathom into conscious and unconscious processes and their neural underpinnings requires at least a dissociation of phenomenology from the actual brain-states underlying our mental conditions. Perception and cognition emerge from processes only partially depending on sensory stimulation and bottom-up neural pathways. An illustrative example is the so-called multistable perception, discussed below.

Regarding *the second hypothesis* it is noted that the unconscious processes preceding feelings were called by the leading investigator in this area, Antonio Damasio, *emotions*. The crucial role of feelings and emotions in homeostasis has been elucidated by several investigators. As an example, the feelings associated with hunger and thirst are discussed later in this article. By generalizing the role of emotions in homeostasis, it is natural to speculate that *emotions capture the body's state in the reactive phase that follows internal as well as external changes*. An example of an internal change is provided by the spontaneous neural oscillations giving rise to an emotionally competent memory. If this further generalization of the notion of emotions is indeed correct, then, every mental function is accompanied by an appropriate emotion, which depending on the circumstances, may or may not become conscious, i.e., may or may not be expressed as a feeling. Taking into consideration the fundamental role of emotions in homeostasis, it follows that emotions are of higher biological value than other types of unconscious functions. In this sense, it is not surprising that, for example, in Capgras's syndrome, emotions override mechanisms related to perception and other cognitive processes.

## 2 The implications of multi-stable perception regarding the nature of awareness

Perception is generative. Visual perception, for instance, is achieved via both the stimulation of sensory cells of retina, as well as via the solution of multiscale *inverse problems*. Specifically, the brain, using as data the distribution of the photons emitted from the sensory input, reconstructs, via backpropagation, a *mental image* of a percept. Namely, it “computes” effective synaptic updates by using feedback connections to constrain neuronal activities, whose locally computed differences encode backpropagation-like error signals. This highly complicated process begins with the reconstruction of constituents of the percept, achieved via the solution of *local* inverse problems, such as the reconstruction and context-related-optimization of, orientation, color, and motion, at early stages of a given percept. Computations are performed by hierarchically organized specific *local neural circuits*, located in different parts of the brain. The solution of each of these inverse problems gives rise to the unconscious reform of specific ingredients of the percept. If there exists sufficient amount of incoming energy entering the retina, then there will occur the simultaneous excitation of several of the local neural circuits *associated* with aspects of this percept, giving rise to *global activation*. The synchronous activation of these interconnected local neural circuits results in the integration of the solutions of the local inverse problems, which ultimately yields awareness, taking the form of a mental image.

**Figure 1:**
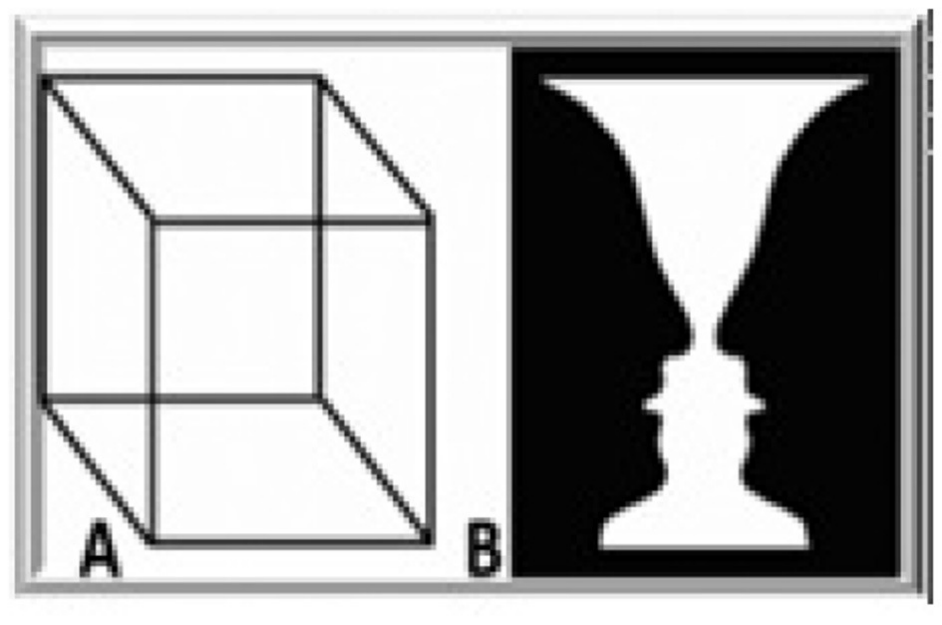
Ambiguous figures (Necker cube and vase-and-faces).

The human brain, although renowned for its awesome “computational” powers, lapses into profound confusion when it receives conflicting views of the visual world. Consider, for example, the so-called Ambiguous Figures presented in Figure 1 (Necker Cube and Vase-and-Faces). The optical sensory input to the visual system remains unchanged, and yet the resulting perceptual interpretation vacillates, over time, between alternative views—a behavior called “perceptual bistability.” The emerging fluctuations presumably occur because the brain is receiving conflicting ambiguous information about the nature of this percept at a given location in visual space. Faced with such ambiguity, the brain fluctuates, over time, between different neural states (Blake and Logothetis, 2002; Leopold and Logothetis, 1999; Logothetis, 1998). Although the input to the sensory system remains the same, the unconscious and conscious processes taking place in the organizational continuum, are changing.

**Figure 2:**
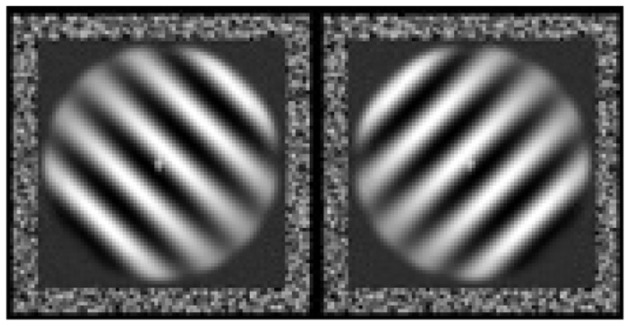
Bistability.

Bistability can be also induced by presenting two different images in each eye, for example, a left-tilted grating on the left, and a right-tilted on the right (see Figure 2). “Binocular rivalry” is an excellent example of perceptual bistability that can be used in combined psychophysical and electrophysiological experiments in trained non-human-primates (NHP) (Logothetis, 1998). Research in this domain established that the neural mechanisms underlying the awareness of a visual pattern during rivalry are distributed over the entire visual pathway, with the percentage of perception-reflecting neurons increasing as one moves from the primary visual cortex into the areas of the temporal lobe. In contrast to the traditional beliefs that rivalry is a form of competition between the two eyes involving reciprocal suppression of retinal inputs, the aforementioned studies demonstrated that rivalry most likely reflects alternation of conscious and unconscious stimulus-representations that are instantiated in the firing patterns of multi-size groups of neurons in different cortical areas. Importantly, the successive periods of visibility and invisibility of a pattern—which are usually referred to as the pattern's (or the eye's) *dominance* and *suppression* phases— are stochastic rather than resulting from the chaotic behavior of a neural dynamical system (Lehky, 1995). The average dominance and suppression periods vary both with subject and with stimulus type. Yet, when individual phase-durations are normalized, e.g. expressed as fractions of their mean, their distribution can be very well approximated by gamma functions, the parameters of which show considerable inter-subject similarity for both humans and NHP (Levelt, 1965).

Interestingly, an increase of the stimulus strength (e.g. luminance-contrast of the gratings) in one eye increases the predominance of this stimulus by decreasing the contralateral eye's mean dominance (Logothetis, 1999). The mean dominance of the eye receiving the stronger stimulus remains unaffected. In other words, increasing the strength of one stimulus decreases the unconscious periods of the stimulus, rather than increasing the duration of the conscious one; this suggests that neural networks underlying conscious perception are different from those involved in the inhibition of a competitive pattern.

It is worth noting that the polymath Hermann von Helmholtz was aware of the lack of a one-to-one correspondence between a percept and its mental image. In his book, *Physiological Optics*, he used the term “unconscious inference” to characterize how our visual apparatus, by using unconscious processes, “computes” the *best interpretation* compatible with incoming sensory data. Remarkably, eight centuries earlier, the Arab scientist Abu Ali al Hasan (965–1040), known in the West as Alhazen, also postulated that our brain, without us being aware, jumps to conclusions beyond its available sensory data. It is fascinating that Alhazen also called this process “unconscious inference” and noted that illusions are generated via this process.

## 3 The importance of emotions

According to the philosopher Gregory Vlastos, Plato was decisively influenced by the power of mathematics, especially after meeting on a visit to Sicily the mathematically knowledgeable philosopher Archytas (Vlastos, 1988). This influence becomes clear by the emphasis placed by Plato on reason, which is “the calculating element of the soul.” According to Plato, reason seeks knowledge and at the same time analyses the short-term as well as and long-term consequences of a given action. The calculating part of the soul corresponds to today's consciousness.

In contrast to Plato's analysis, the two hypotheses discussed in this article emphasize the indispensable role of emotions in particular, and of unconscious processes in general. Emotions are *evolutionarily* older than reason and hence are more important for our survival; in particular, they are crucial for *homeostasis*. In this connection, it is noted that in the same way that the formation of stars and planets in our universe requires that certain specific physical constants are in a narrow range (Rees, 2001), life in an organism is maintained only when several conditions are simultaneously met in its interior. The process of maintaining a state necessary for survival and reproduction is called homeostasis. This term, which derives from the Greek words homeo (similar) and stasis (equilibrium), was introduced by the Harvard physiologist Walter Cannon (1871–1945). The predecessor of this notion is the concept of milieu interieur (internal environment), advocated by the French physiologist Claude Bernard (1813–1878), which states that the internal environment exists in a state of stability. According to Bernard, the purpose of a variety of biological mechanisms is to ensure that any deviation from equilibrium is efficiently addressed so that the organism returns to a perfect state. A more dynamic and accurate notion of homeostasis arose from the ideas of Elie Metchnikoff (1845–1916) who was awarded the Nobel Prize in Medicine in 1908. By employing analogical thinking, this pioneer zoologist implemented Darwin's idea of *antagonism* inside an organism: the antagonistic efforts of different cell lines to evolve, develop, and differentiate, create a state of *dynamic disharmon*y. Bernard's state represents only an *ideal* situation.

Achieving homeostasis involves reflexes as well as mostly unconscious processes. However, the positive impact of consciousness should not be underestimated. Actually, the diverse and overlapping utilization of the triptych, reflexes-unconscious processes-consciousness, is of paramount importance for maintaining homeostasis. Recent developments in neuroscience have allowed researchers to begin to elucidate the precise neuronal mechanisms that control basic homeostatic processes. For example, it is now possible to begin answering the question of, “how do we become hungry?” This question is related to the need of the organism to maintain *energy balance*. It is now known that if fat stores fall, the level of the hormone *leptin*, which is secreted by adipose cells, also falls. This activates a certain type of neurons called agouti-related peptide (AgPR). These neurons, which are located at the base of the hypothalamus, initiate a complicated process that promotes *energy restoration*. This is achieved via two basic mechanisms: first, reducing expenditure, and second, inducing the unpleasant feeling of hunger (Lowell, 2019). The reduction of energy expenditure following the activation of AgPR neurons occurs via an unconscious process that causes the inhibition of the activity of the sympathetic nervous system and the reduction of thermogenesis in brown adipose tissue. The precise neuronal pathways which, starting with the activation of AgPR neurons, finally give rise to the *awareness* of the unpleasant feeling of hunger, are currently under investigation. Apparently, these pathways involve unconscious processes in the midbrain and the hypothalamus, which are followed by conscious processes involving the insula. A specific pathway has already been delineated: it involves AgPR neurons to *melanocortin IV* receptors in the *paraventricular* nucleus of the hypothalamus, and then to neurons in the lateral *branchial nucleus* located at the boarder of pons and the midbrain (Berrios et al., 2021).

Incidentally, the time scale associated with the awareness of hunger, namely the time from the stimulation of AgPR neurons to the time of seeking food, is of the order of a minute, which is much longer than the scales occurring in perception, which are of the order of one-third of a second. Apparently, this is due to the involvement of many synapses, as well as to the action of several neuromodulators whose indirect action is much slower than the usual direct action in the ionic gates of the standard neurotransmitters (Berrios et al., 2021).

In the opposite situation, where leptin is raised, another type of neurons is activated, called *proopiomelanocortin*. These neurons, which are also located at the base of hypothalamus, release a particular hormone, the α*-melanocyte-stimulating* hormone, which promotes weight loss.

Progress has also been made toward answering the question of, “how we become thirsty?” In brief, the brain senses water loss by detecting blood changes of the hormone *angiotensin* II, and then restores balance via two basic mechanisms: first, reducing excretion, and second causing thirst. Reduction of excretion is achieved via unconscious changes in the activity of the *vasopressin* neurons, whereas thirst is induced via the activation of specific hypothalamic neurons, called median preoptic nucleus (MnPO) (Gizowski and Bourque, 2018).

The activation of the AgRP and MnPO neurons is a necessary and sufficient condition for the occurrence of hunger and thirst. Thus, the circuits formed by these neurons provide the neural basis that gives rise to the *feelings* of hunger and thirst. This discussion suggests that a feeling is a specific conscious form of a subset of unconscious processes.

Why do we use the term “subset”? We believe that this term is justified by noting that, in the above examples, the full set of the relevant unconscious processes not only involves those processes that finally give rise to the awareness of hunger and thirst, but also processes that *never* become conscious. The latter are the processes that yield a decrease in energy expenditure, as well as reduction in water excretion. Thus, only the subset of unconscious processes related to hunger and thirst finally gives rise to consciousness, namely the feelings of hunger and thirst.

Homeostatic emotions provide the response of the organism to a detected homeostatic disturbance in the structure or function of the viscera, which is caused by the change of a specific physiological quantity. Thus, *emotions should include the full set of unconscious processes preceding a feeling*. For example, the unconscious processes involved in the decrease of expenditure should be part of the emotion associated with the feeling of hunger. These remarks are consistent with ideas of Damasio (2018) and highlight further the fact that consciousness reveals only a small part of reality (Fokas, 2024).

## Data Availability

The original contributions presented in the study are included in the article/supplementary material, further inquiries can be directed to the corresponding author.
